# Comparing Generative Artificial Intelligence and Mental Health Professionals for Clinical Decision-Making With Trauma-Exposed Populations: Vignette-Based Experimental Study

**DOI:** 10.2196/80801

**Published:** 2025-10-14

**Authors:** Katherine E Wislocki, Sabahat Sami, Gahl Liberzon, Alyson K Zalta

**Affiliations:** 1 Department of Psychological Science, University of California, Irvine, 4201 Social and Behavioral Sciences Gateway, Irvine, CA, 92697, United States, 1 949-824-5574

**Keywords:** generative artificial intelligence, trauma, mental health professionals, diagnosis, treatment

## Abstract

**Background:**

Trauma exposure is highly prevalent and associated with various health issues. However, health care professionals can exhibit trauma-related diagnostic overshadowing bias, leading to misdiagnosis and inadequate treatment of trauma-exposed populations. Generative artificial intelligence (GAI) models are increasingly used in health care contexts. No research has examined whether GAI demonstrates this bias in decision-making and how rates of this bias may compare to mental health professionals (MHPs).

**Objective:**

This study aimed to assess trauma-related diagnostic overshadowing among frontier GAI models and compare evidence of trauma-related diagnostic overshadowing between frontier GAI models and MHPs.

**Methods:**

MHPs (N=232; mean [SD] age 43.7 [15.95] years) completed an experimental paradigm consisting of 2 vignettes describing adults presenting with obsessive-compulsive symptoms or substance abuse symptoms. One vignette included a trauma exposure history (ie, sexual trauma or physical trauma), and one vignette did not include a trauma exposure history. Participants answered questions about their preferences for diagnosis and treatment options for clients within the vignettes. GAI models (eg, Gemini 1.5 Flash, ChatGPT-4o mini, Claude Sonnet, and Meta Llama 3) completed the same experimental paradigm, with each block being reviewed by each GAI model 20 times. Mann-Whitney *U* tests and chi-square analyses were used to assess diagnostic and treatment decision-making across vignette factors and respondents.

**Results:**

GAI models, similar to MHPs, demonstrated some evidence of trauma-related diagnostic overshadowing bias, particularly in Likert-based ratings of posttraumatic stress disorder diagnosis and treatment when sexual trauma was present (*P*<.001). However, GAI models generally exhibited significantly less bias than MHPs across both Likert and forced-choice clinical decision tasks. Compared to MHPs, GAI models assigned higher ratings for the target diagnosis and treatment in obsessive-compulsive disorder vignettes (*r_b_*=0.43‐0.63; *P*<.001) and for the target treatment in substance use disorder vignettes (*r_b_*=0.57; *P*<.001) when trauma was present. In forced-choice tasks, GAI models were significantly more accurate than MHPs in selecting the correct diagnosis and treatment for obsessive-compulsive disorder vignettes (χ²_1_=48.84‐61.07; *P*<.001) and for substance use disorder vignettes involving sexual trauma (χ²_1_=15.17‐101.61; *P*<.001).

**Conclusions:**

GAI models demonstrate some evidence of trauma-related diagnostic overshadowing bias, yet the degree of bias varied by task and model. Moreover, GAI models generally demonstrated less bias than MHPs in this experimental paradigm. These findings highlight the importance of understanding GAI biases in mental health care. More research into bias reduction strategies and responsible implementation of GAI models in mental health care is needed.

## Introduction

Trauma exposure is associated with a range of health-related issues, including physical health problems [1,2] and mental health problems [3,4]. Owing to the potential impact of trauma exposure on individual health and well-being, assessing trauma exposure history is an essential part of mental health assessment [5,6]. Trauma exposure is quite common, with some estimates suggesting that around 90% of individuals experience trauma during their lifetime. However, the incidence of trauma-related mental health problems following trauma exposure is much less common [3]. As a result, many individuals seeking mental health care are likely to report a history of trauma, even if they are not presenting with trauma-related symptoms.

Previous research has indicated that trauma-related diagnostic overshadowing bias occurs when health care professionals prioritize an individual’s trauma history in diagnostic and treatment decision-making, rather than focusing on the individual’s primary symptoms [7,8]. Experimental research has shown that mental health professionals (MHPs) are more likely to provide a posttraumatic stress disorder (PTSD) diagnosis and recommend PTSD-specific treatment for individuals with a trauma history, even when the trauma exposure occurs after the onset of primary symptoms and those symptoms do not warrant a PTSD diagnosis or PTSD treatment [7,8]. The consequences of trauma-related diagnostic overshadowing bias are likely to be significant and far-reaching for both trauma-exposed individuals and the mental health care system. By prioritizing trauma history over current symptoms, MHPs risk misdiagnosing and providing inadequate treatments to trauma-exposed individuals. This can lead to individuals receiving diagnoses and treatments that do not address their presenting concerns, potentially delaying their ability to receive optimal care and exacerbating their primary symptoms [9,10].

An increasing body of research has used generative artificial intelligence (GAI) models to assist with health care, including the use of GAI for diagnosis [11], treatment planning [12,13], treatment delivery [14,15], and other clinical care tasks [16]. Although GAI has existed for some time, the development of more advanced models has led to greater proliferation of GAI technologies in health care. There is a growing body of literature that not only evaluates the use of GAI in clinical decision-making but also compares clinical decision-making across MHPs and GAI models in a variety of tasks [17]. For example, a recent clinical trial demonstrated higher rates of diagnostic accuracy between large language models and physician samples in response to clinical vignettes [17]. Additional research has shown that GAI can be used to support and improve clinical decision-making among health care professionals [18]. Within the context of mental health, several studies have compared the clinical decision-making across MHPs and GAI [19-22]. These studies have found that GAI models can perform comparably to, and in some cases better than, MHPs in identifying diagnoses and recommending evidence-based treatments [19-22]. While GAI may demonstrate a high level of performance on clinical decision-making tasks, research has also raised important concerns about bias and limitations in GAI clinical decision-making [21,23].

Errors in clinical decision-making from GAI models in health care applications can be quite harmful [24,25]. Recent research indicates that individuals may use GAI to “help manage their emotional and mental health” [26]. As GAI models are increasingly scaled for diverse health care contexts, greater attention is being paid to the potential biases and limitations associated with their use in health care [25,27-29]. As GAI is developed and trained using inherently biased data, this may result in biased clinical decision-making being scaled to real-world environments [27,29]. For example, previous work has demonstrated significant biases in GAI responses related to clinical decision-making, including gender-related bias in diagnosing comorbid mental health conditions [21] as well as racial biases in diagnosis [29] and emergent clinical decision-making for individuals experiencing mental health crises [30]. Assessing the potential for bias in decision-making by GAI models is critical for reducing potential harms associated with this technology. However, there is a dearth of research focused on examining bias and other errors in clinical decision-making involving trauma-exposed populations by GAI models.

This study aimed to fill this gap by conducting a secondary data analysis of a prior study on trauma-related diagnostic overshadowing bias among MHPs [8]. Specifically, the study assessed trauma-related diagnostic overshadowing among GAI models and compared evidence of trauma-related diagnostic overshadowing between GAI models and MHPs. This study supports the growing body of literature focused on evaluating bias in artificial intelligence, as it relates to mental health care. The results from this research may help inform the development, evaluation, and implementation of GAI in mental health care for trauma-exposed populations.

## Methods

### Participants

#### Mental Health Professionals

In the initial study, the sample of MHPs was recruited from professional listservs and networks [8]. MHPs (N=232) reported a mean (SD) age of 43.73 (15.95) years (71.98% female-identifying). The sample mostly comprised doctoral-level (n=115, 53.99%) and master’s-level (n=90, 42.25%) professionals, primarily in the fields of clinical psychology (n=129, 60.56%) and social work (n=67, 31.46%). MHPs had a mean (SD) of 15.41 (13.73) years of experience.

#### GAI Models

A total of 4 large-scale GAI models were used, that is, Llama 3.0 [31], ChatGPT-4o mini [32], Gemini 1.5 Flash [33], and Claude Sonnet [34], to assess trauma-related diagnostic overshadowing among GAI models (each model is hereafter referred to by the name of the model developer). These models were selected as they had demonstrated high-level performance across a range of diverse benchmarks focused on general knowledge, reasoning, instruction following, and language comprehension (eg, Massive Multitask Language Understanding) [35,36]. These models have been used in past research to assess clinical decision-making capabilities [18,20,37-39].

### Procedures

In the initial study, MHPs were randomized to 1 of 8 experimental blocks in which each block contained 2 brief vignettes. Trauma exposure was present in only 1 of the vignettes. The first vignette depicted an adult experiencing obsessive-compulsive disorder (OCD), and the second vignette depicted an adult experiencing substance use disorder (SUD) (Multimedia Appendix 1). Cases contained enough information to support the target diagnoses and treatment of OCD and SUD but did not contain information to warrant diagnosis or treatment of PTSD (Multimedia Appendix 2). Furthermore, when trauma exposure was present, it was explicitly indicated to have occurred following the onset of the primary presenting symptoms. For the vignettes containing trauma exposure, the type of trauma exposure was counterbalanced across conditions such that vignettes presented either physical trauma exposure (ie, single incident of a “serious” motor vehicle accident with lingering physical injuries) or sexual trauma exposure (ie, single incident of sexual assault by a coworker during a business trip). The sex of the individual in the vignette was also counterbalanced (ie, a female-identifying individual was the focus of a vignette and a male-identifying individual was the focus of the other vignette).

To ensure reliability, each GAI model was queried 20 times using the same experimental vignettes delivered to MHPs (ie, 8 experimental blocks containing 2 vignettes each) [22]. To make it as similar as possible to that delivered to MHPs, each iteration was performed in 1 prompt that combined the vignettes and questions for each experimental block. Across cases, this resulted in a total of 160 responses to vignettes containing sexual trauma, 160 responses to vignettes containing physical trauma, and 320 responses to vignettes containing no trauma. Previous work has demonstrated that GAI models do not update model weights during inference, resulting in little carryover of memory from session to session [40]. To reduce the potential impact of model memory on output, all prompts were entered into each GAI model through the web-based implementations of each GAI model using a new instance of a Google Chrome incognito browser for each iteration and model. Prompts were limited to vignettes, questions, and prompt instructions alone to match as closely as possible the information presented to MHPs. Prompts, containing the vignettes, questions, and instructions, were entered once per session for each model. All vignettes and prompts can be found in Multimedia Appendix 1. To mirror the paradigm completed by MHPs, hyperparameters (such as temperature, which can affect the randomness of model responses, or maximum length, which can affect the length of responses) were not manipulated. All GAI models were prompted in January 2025.

After reviewing each vignette, both MHPs and GAI models responded to diagnostic and treatment questions related to the vignettes. For GAI models, each question included additional prompt information to ensure that the corresponding response matched the format of the question (eg, “please select the most clinically appropriate treatment model and include that below with no other information” for forced-choice questions).

### Measures

After reviewing each vignette, respondents rated the likelihood that the individual would meet diagnostic criteria for a range of mental health disorders using a 7-point Likert scale (1=“not at all likely,” 7=“extremely likely”). Diagnostic options included OCD, adjustment disorder, major depressive disorder, generalized anxiety disorder, PTSD, and SUD. Trauma-related diagnostic overshadowing for these likelihood ratings was defined as lower ratings for the target diagnosis (ie, OCD or SUD) or higher ratings for a PTSD diagnosis when trauma exposure was present versus absent. Respondents were then asked to select the primary diagnosis they would assign from the list of diagnoses. Trauma-related diagnostic overshadowing in diagnosis selection was indicated when respondents were less likely to select the target diagnosis (OCD or SUD) or more likely to select a PTSD diagnosis in trauma-present vignettes compared to trauma-absent vignettes.

Next, respondents rated the clinical appropriateness of specific mental health treatments for the individuals using a 7-point Likert scale (1=“extremely inappropriate”, 7=“extremely appropriate”). Treatment options included dialectical behavior therapy, cognitive processing therapy (CPT), exposure and response prevention (ERP), motivational interviewing (MI), and psychodynamic psychotherapy, with the option to enter an alternative treatment. Trauma-related diagnostic overshadowing for these treatment ratings was defined as lower appropriateness ratings for the target treatment (ERP for OCD case; MI for SUD case) or higher ratings for a PTSD-specific treatment (CPT) when trauma exposure was present versus absent. Finally, respondents selected the most appropriate treatment from the list of treatments. Trauma-related diagnostic overshadowing in treatment selection was indicated when respondents were less likely to select the target treatment (ERP or MI) or more likely to select the PTSD treatment (CPT) in response to vignettes where trauma exposure was present, compared to when trauma exposure was absent.

### Data Analysis

Normality in Likert-based data was assessed using *z* tests based on skewness and kurtosis values. These tests indicated deviations from normality across Likert-based response variables for both cases. Therefore, to establish whether GAI models demonstrated bias (Aim 1), Mann-Whitney *U* tests were used to compare Likert ratings for target diagnosis, target treatment, PTSD diagnosis, target treatment, and PTSD treatment across no trauma and trauma conditions (ie, no trauma vs physical trauma, no trauma vs sexual trauma) as well as physical trauma versus sexual trauma conditions for each case (ie, OCD and SUD). Chi-square analyses were used to compare forced-choice selection of target diagnosis, PTSD diagnosis, target treatment, and PTSD treatment across no trauma and trauma conditions (ie, no trauma vs physical trauma, no trauma vs sexual trauma) as well as physical trauma versus sexual trauma conditions for each case. To compare the rates of bias across GAI models and MHPs (Aim 2), Mann-Whitney *U* tests were used to compare Likert ratings for target diagnosis, PTSD diagnosis, target treatment, and PTSD treatment for vignettes including trauma (ie, vignettes including physical trauma or sexual trauma) across each case. Chi-square analyses were used to compare forced-choice selection of target diagnosis, PTSD diagnosis, target treatment, and PTSD treatment for vignettes including trauma (ie, vignettes including physical trauma or sexual trauma) across each case. Effect sizes were calculated for comparison of Likert data using rank-biserial correlations [41]. Bootstrapped CIs are provided for rank-biserial correlations. Effect sizes (*r_b_*) are interpreted using Cohen’s conventions: small ≥0.10, medium ≥0.30, and large ≥0.50 [42]. A Bonferroni correction was applied to adjust for the number of tests, resulting in an adjusted α value of .001. All analyses were performed using R version 4.3.1 [43].

### Ethical Considerations

Procedures for the initial study received approval from the Institutional Review Board (IRB) at the University of California, Irvine (IRB #448). Mental health professionals provided informed consent as part of the initial study. More information on the initial study can be found in past work [8]. No additional approval was required for secondary data analysis.

## Results

### Do GAI Models Demonstrate Bias?

#### Results From Likert Ratings

Likert ratings of diagnosis and treatment options from GAI models, as well as results from Mann-Whitney *U* tests comparing within-model Likert ratings for each response choice can be found in Table 1.

**Table 1.** Likert ratings of diagnosis and treatment options by GAI models^a^.

	Target^b^			PTSD^c^		
Model	No trauma, mean (SD)	Physical trauma, mean (SD)	Sexual trauma, mean (SD)	No trauma, mean (SD)	Physical trauma, mean (SD)	Sexual trauma, mean (SD)
OCD case—diagnosis ratings^d^						
ChatGPT	6.88 (0.33)^e^	6.98 (0.16)^e^	6.90 (0.30)^e^	1.00 (0.00)	3.33 (0.66)^e^	5.13 (0.56)^f^
Claude	7.00 (0.00)	7.00 (0.00)	7.00 (0.00)	1.00 (0.00)	3.93 (0.35)^e^	5.68 (0.62)^f^
Gemini	6.64 (0.48)^e^	6.75 (0.44)^e^	6.98 (0.16)^f^	1.00 (0.00)	5.15 (0.36)	6.00 (0.00)
Llama	6.79 (0.41)^e^	6.93 (0.27)^e^	6.28 (0.51)^f^	1.01 (0.11)^e^	4.28 (0.45)^f^	5.80 (0.41)^g^
OCD case—treatment ratings^d^						
ChatGPT	7.00 (0.00)	7.00 (0.00)	7.00 (0.00)	2.69 (0.56)^e^	3.15 (0.48)^f^	3.85 (0.48)^g^
Claude	7.00 (0.00)	7.00 (0.00)	7.00 (0.00)	2.45 (0.50)^e^	3.15 (0.48)^f^	4.50 (0.88)^g^
Gemini	7.00 (0.00)	7.00 (0.00)	7.00 (0.00)	4.00 (0.00)	4.63 (0.59)	6.00 (0.00)
Llama	7.00 (0.00)	7.00 (0.00)	6.68 (0.47)	2.45 (0.50)^e^	3.10 (0.44)^f^	5.20 (1.29)^g^
SUD case—diagnosis ratings^d^						
ChatGPT	7.00 (0.00)	7.00 (0.00)	7.00 (0.00)	1.00 (0.00)	3.45 (0.60)^e^	6.10 (0.30)^f^
Claude	7.00 (0.00)	7.00 (0.00)	7.00 (0.00)	1.13 (0.33)^e^	5.00 (0.00)	5.80 (0.88)^f^
Gemini	7.00 (0.00)	7.00 (0.00)	7.00 (0.00)	1.00 (0.00)	4.03 (0.53)	6.00 (0.00)
Llama	7.00 (0.00)	7.00 (0.00)	7.00 (0.00)	1.46 (0.50)^e^	5.58 (0.50)^f^	6.20 (0.41)^g^
SUD case—treatment ratings^d^						
ChatGPT	7.00 (0.00)	7.00 (0.00)	7.00 (0.00)	2.63 (0.51)^e^	3.00 (0.51)^f^	5.00 (0.93)^g^
Claude	7.00 (0.00)	7.00 (0.00)	7.00 (0.00)	2.63 (0.49)^e^	4.95 (0.22)^f^	5.98 (0.16)^g^
Gemini	7.00 (0.00)	7.00 (0.00)	7.00 (0.00)	2.95 (0.39)^e^	4.28 (0.45)^f^	6.00 (0.00)
Llama	6.99 (0.11)^e^	7.00 (0.00)	6.95 (0.22)^e^	2.26 (0.47)^e^	4.63 (0.59)^f^	5.10 (0.44)^g^

a Values for target diagnosis and treatment closer to a score of 7 and values for PTSD diagnosis and treatment closer to a score of 1 reflect less trauma-related diagnostic overshadowing bias. Mann-Whitney *U* tests could not be conducted when there was no variance in responses.

b The target column reflects mean ratings for target diagnosis or treatment for each case.

c The PTSD column reflects mean ratings for PTSD diagnosis or treatment for each case.

d Different superscripts (ie, e, f, and g) denote significantly within-model differences in ratings at the Bonferroni-corrected significance level (*P *< .001) through a Mann-Whitney *U* test

The mean ratings for the target diagnosis (range 6.28‐7.00) and target treatment (range 6.68‐7.00) were quite high across all models, regardless of case type and presence of trauma exposure. This reflects evidence that GAI models tended to demonstrate minimal to no bias in target diagnosis and treatment ratings. Llama provided significantly lower ratings of OCD diagnosis and OCD treatment when sexual trauma was present in the OCD case compared to when no trauma was present (Table 1), indicating evidence of bias. Unexpectedly, Gemini demonstrated significantly greater ratings of OCD diagnosis in the OCD case when sexual trauma was present, compared to when it was absent (Table 1). However, the overall magnitude of difference in ratings was small (less than 1 point on the scale) for both Llama and Gemini across cases, indicating slight evidence of bias in within-model target diagnosis and target treatment ratings.

The mean ratings for PTSD diagnosis (range 1.00‐6.20) and PTSD treatment (range 2.26‐6.00) varied across all models. There was consistent evidence of bias in comparing Likert ratings of PTSD diagnosis and treatment in vignettes with and without trauma exposure, such that all models across both cases demonstrated significantly higher ratings of PTSD diagnosis and PTSD treatment when trauma exposure was present, compared to when it was absent (Table 1). Furthermore, evidence of bias was stronger for vignettes containing sexual trauma versus physical trauma, as all models tended to assign greater ratings of PTSD diagnosis and PTSD treatment when sexual trauma was present, compared to when physical trauma was present (Table 1). Notably, the magnitude of differences in PTSD diagnosis ratings across vignettes with and without trauma was substantial (range of 2.33-5.00 points on the 7-point Likert scale), providing a clear indication of bias. The magnitude of differences in PTSD treatment ratings across vignettes with and without trauma was smaller (range of 0.37-3.35 points on the 7-point Likert scale), yet still indicative of bias.

#### Results From Forced Choice Ratings

Forced-choice selection of diagnosis and treatment options can be found in Multimedia Appendix 3. ChatGPT, Gemini, and Claude demonstrated no evidence of bias by correctly selecting the target diagnosis and treatment 100% of the time. Llama correctly selected the target diagnosis and treatment in 95% of vignettes for the SUD case. However, Llama demonstrated evidence of bias in the OCD case, selecting the target diagnosis and treatment 62.50% and 65% of the time, respectively, and the PTSD diagnosis and treatment 37.50% and 35% of the time, respectively. Notably, the evidence of bias in diagnosis and treatment selection in Llama responses was present only in OCD vignettes that contained sexual trauma (Multimedia Appendix 3).

### Do GAI Models Demonstrate Less Bias Than MHPs?

#### Results From Likert Ratings

Results from the analysis of Likert-based ratings of diagnosis and treatment from GAI and MHPs are found in Table 2.

**Table 2.** Likert ratings of trauma vignettes from GAI models and mental health professionals^a^*.*

	Physical trauma				Sexual trauma			
Outcome	MHP^b^, mean (SD)	GAI^c^, mean (SD)	*U*	*r*_b_ (95% CI)	MHP^b^, mean (SD)	GAI^c^, mean (SD)	*U*	r_b_ (95% CI)
OCD^d^ case								
Target diagnosis	5.75 (1.61)	6.91 (0.28)	6600^e^	0.53 (0.39-0.65)	5.86 (1.37)	6.79 (0.43)	6749^e^	0.43 (0.29-0.55)
Target treatment	5.69 (1.83)	7.00 (0.00)	—^f^	—	5.12 (2.01)	6.91 (0.27)	6698^e^	0.63 (0.50-0.75)
PTSD^g^ diagnosis	4.41 (1.89)	4.17 (0.81)	3624	0.13 (0.01-0.30)	5.78 (1.26)	5.65 (0.56)	3700^h^	0.14 (0.01-0.33)
PTSD treatment	3.92 (1.97)	3.51 (0.82)	3316	0.11 (0.01-0.28)	4.96 (1.92)	4.89 (1.13)	3716	0.09 (0.00-0.26)
SUD^i^ case								
Target diagnosis	6.89 (0.32)	7.00 (0.00)	—	—	6.74 (0.60)	7.00 (0.00)	—	—
Target treatment	6.15 (1.49)	7.00 (0.00)	—	—	5.69 (1.85)	6.99 (0.11)	5489^e^	0.57 (0.44-0.69)
PTSD diagnosis	5.10 (1.07)	4.51 (0.95)	2842.5^e^	0.25 (0.11-0.38)	5.56 (1.30)	6.03 (0.53)	4758^j^	0.19 (0.02-0.40)
PTSD treatment	4.24 (1.63)	4.21 (0.87)	3889.5	0.01 (0.00-0.21)	4.90 (1.87)	5.52 (0.70)	3748.5	0.09 (0.01-0.28)

a Mann-Whitney *U* tests could not be conducted when there was no variance in responses. Effect sizes for the differences in ratings between MHP and GAI are calculated using rank biserial correlations (rb) [41]. Effect sizes (rb) are interpreted using Cohen’s conventions: small ≥0.10, medium ≥0.30, and large ≥0.50 [42]. Ratings for target diagnosis and treatment closer to a score of 7 and ratings for PTSD diagnosis and treatment closer to a score of 1 reflect less trauma-related diagnostic overshadowing bias.

b MHP: mental health professionals.

c GAI: generative artificial intelligence.

d OCD: obsessive-compulsive disorder.

e *P*<.001

f not available.

g PTSD: posttraumatic stress disorder.

h *P*<.05.

i SUD: substance use disorder.

j *P*<.01.

Compared to MHPs, GAI models tended to exhibit less bias by assigning significantly greater target diagnosis and target treatment ratings for both the OCD case and SUD case when trauma exposure was present (Table 2). Across both cases, effect sizes for comparisons of target diagnosis and treatment ranged from moderate to large (Table 2). There were no significant differences between MHPs’ and GAI models’ ratings of PTSD diagnosis and PTSD treatment for both cases using the Bonferroni-corrected significance level (*P*=.001), with the exception that compared to MHPs, GAI models provided significantly lower ratings of PTSD diagnosis in the SUD case when physical trauma was present (Table 2).

#### Results From Forced Choice Ratings

Percentages of forced-choice selections of diagnosis and treatment are included in Table 3.

**Table 3.** Forced-choice selections from GAI models and mental health professionals^a^*.*

	Physical trauma			Sexual trauma		
Outcome, n/N (%)	MHP^b^	GAI^c^	Chi-square (*df*)	MHP	GAI	Chi-square (*df*)
OCD^d^						
Target diagnosis	35/55 (63.64)	160/160 (100)	59.91^e^ (1)	27/59 (45.76)	145/160 (90.63)	48.84^e^ (1)
Target treatment	34/54 (62.96)	160/160 (100)	61.07^e^ (1)	25/58 (43.10)	146/160 (91.25)	55.54^e^ (1)
PTSD^f^ diagnosis	10/55 (18.18)	0/160 (0)	26.55^e^ (1)	25/59 (42.37)	15/160 (9.38)	29.27^e^ (1)
PTSD treatment	11/54 (20.37)	0/160 (0)	30.31^e^ (1)	17/58 (29.31)	14/160 (8.75)	13.12^e^ (1)
SUD^g^						
Target diagnosis	51/55 (92.73)	160/160 (100)	8.21^h^ (1)	42/50 (84)	158/160 (98.75)	15.17^e^ (1)
Target treatment	35/55 (63.64)	160/160 (100)	59.91^e^ (1)	19/50 (38)	158/160 (98.75)	101.61^e^ (1)
PTSD^f^ diagnosis	2/55 (3.63)	0/160 (0)	2.59 (1)	8/50 (16)	2/160 (1.25)	15.17^e^ (1)
PTSD treatment	4/55 (7.27)	0/160 (0)	8.21^h^ (1)	11/50 (22)	2/160 (1.25%)	24.79^e^ (1)

a Values of 100% for target diagnosis and treatment and 0% for PTSD diagnosis and treatment reflect less trauma-related diagnostic overshadowing bias.

b MHP: mental health professionals.

c GAI: generative artificial intelligence.

d OCD: obsessive-compulsive disorder.

e *P*<.001.

f PTSD: posttraumatic stress disorder.

g SUD: substance use disorder.

h *P*<.01.

For the OCD case, there was consistent evidence that GAI models demonstrated less bias than MHPs across all outcomes (Table 3). MHPs selected the correct OCD diagnosis and treatment approximately 64% and 63% of the time in the physical trauma vignettes and approximately 46% and 43% of the time in the sexual trauma vignettes. In contrast, GAI models selected the correct OCD diagnosis and treatment 100% of the time in physical trauma vignettes and 91% of the time in sexual trauma vignettes. For the SUD case, GAI models consistently demonstrated less bias than MHPs in all outcomes for the sexual trauma vignettes (Table 3). In SUD vignettes including physical trauma, GAI models demonstrated less bias only in selecting the target treatment (Table 3). In the SUD case, there were no significant differences between MHPs’ and GAI models’ selection of target diagnosis, PTSD diagnosis, or PTSD treatment in physical trauma vignettes (Table 3). MHPs selected the correct SUD diagnosis and treatment approximately 93% and 64% of the time in the physical trauma vignettes, respectively, and 84% and 38% of the time, respectively, in the sexual trauma vignettes. GAI models assigned the correct diagnosis and treatment 100% of the time in physical trauma vignettes and approximately 99% of the time in sexual trauma vignettes.

## Discussion

This study is the first to evaluate trauma-related diagnostic overshadowing among GAI models and the first to compare rates of trauma-related diagnostic overshadowing between health care professionals and GAI models. From these results, it is clear that GAI models show some evidence of bias. However, the degree to which GAI models demonstrated bias in this study appears to depend on the specific task and model. Specifically, evidence of bias was more apparent when GAI models were asked to rate the likelihood of assigning a PTSD diagnosis and treatment but showed almost no bias when asked to select a primary diagnosis and treatment. Furthermore, there were some notable differences in the rates of bias across GAI models. This was most apparent for forced-choice diagnostic and treatment decision-making, as Llama was responsible for all evidence of bias in forced-choice diagnosis and treatment selection, whereas ChatGPT, Gemini, and Claude correctly selected the appropriate diagnosis and treatment across all vignettes. This suggests that the ways in which GAI models are engineered and trained can affect whether they exhibit bias. Our findings align with past research showing that GAI models demonstrate evidence of a variety of biases that may impact health care [19,21,24,44,45]. Given that GAI is increasingly being used in health care settings [26,46], understanding the conditions that increase biased diagnostic and treatment decision-making and what prompting strategies can be used to reduce bias is an important direction for future research.

Although GAI models were not bias-free, they demonstrated less bias compared to MHPs under many conditions. Past research has demonstrated more accurate clinical decision-making among GAI models, compared to MHPs, across a range of diagnoses [20-22]. In this study, GAI models were more accurate in rating the likelihood of selecting the target diagnosis and treatment across both cases and trauma types. They were also more accurate when selecting a primary diagnosis and treatment for the OCD cases and the SUD case when sexual trauma was present. Though differences were not found in selecting a primary diagnosis and treatment for the SUD case when physical trauma was present (with the exception of target treatment), this is because MHPs showed minimal bias in this case. While these findings may suggest that GAI models may be useful in supporting MHPs engaging in clinical decision-making tasks with trauma-exposed populations, it is critically important to further understand the potential consequences of scaling GAI to support clinical decision-making in mental health care.

Similar to MHPs [7,8], GAI models produce greater bias in decision-making when sexual trauma exposure was present compared to when physical trauma was present. Future research should thoroughly evaluate diagnostic and treatment decisions in response to trauma-exposed populations experiencing a range of different clinical presentations and coming from different demographic backgrounds. In addition, our findings showed that the type of question asked (eg, Likert vs forced-choice) impacted the degree of bias. GAI models can demonstrate significant variability in response to different prompts, and more work is needed to identify and examine how different prompting strategies produce errors in GAI responses (eg, GAI model “hallucinations” or when GAI models produce inaccurate or fabricated information in response to prompts).

While the precise training data and methods used by developers of large-scale GAI models largely remain proprietary, several plausible explanations exist for why GAI models may demonstrate patterns of bias such as trauma-related diagnostic overshadowing. GAI models are trained on vast amounts of internet-based data, which includes both data related to (eg, clinical case vignettes, diagnostic standards, treatment best practices) and unrelated to clinical decision-making (ie, misinformation, pseudoscience) [27]. As noted in prior research, GAI often demonstrates factual knowledge similar to many professionals across various contexts, including in mental health, yet it simultaneously demonstrates difficulties with clinical decision-making, particularly in nuanced or complex situations [21,23]. Furthermore, past research has demonstrated evidence of a range of biases in GAI decision-making as it relates to mental health, including racial bias [30] and gender bias [21]. Given that GAI models are not explicitly taught to engage in the steps of clinical decision-making (eg, differential diagnosis) but instead are trained to predict likely completions of text based on patterns in their training data, they may default to overemphasizing trauma-related outcomes when trauma is mentioned. This aligns with literature indicating that LLMs can exhibit particular cognitive biases (eg, anchoring) [47], particularly when exposed to emotionally salient information such as trauma [48]. Consequently, trauma-related diagnostic overshadowing in GAI models may stem from these models making decisions probabilistically based on the available context and its training data rather than through logic. For example, elevated Likert ratings for PTSD diagnosis and treatment in response to vignettes with sexual trauma reflect the well-established finding that sexual trauma is particularly traumatogenic [49].

Prior research has demonstrated that GAI models may be helpful in supporting physician decision-making [18]. However, there has been little research on how GAI models can support clinical decision-making for mental health care [50]. Given that a portion of MHPs already use GAI models in their work [26], it is particularly important to not only understand if these tools may be helpful in supporting clinical decision-making, but also it is important to understand how these tools may be helpful in supporting clinical decision-making. Research has indicated that artificial intelligence (AI)–assisted diagnosis has affected clinical decision-making processes in other areas of health care, such that health care professionals use AI-derived diagnostic information as a first step, then “work backward” to confirm whether the diagnostic prediction matches available information from other sources (eg, patients, experts, and existing guidelines) [51]. Although GAI has the potential to serve a similar function in mental health care, there are numerous important considerations for using GAI in supporting clinical decision-making in a mental health context. Research has demonstrated that humans are often overconfident in the accuracy of GAI model responses [52], which may potentially result in greater bias in clinical decision-making in instances where GAI models make biased decisions. Past research has indicated that bias from AI can negatively impact individual decision-making both in specific tasks and beyond, as there is evidence that humans “inherit” and “generalize” AI-derived bias to new situations [53]. Given the potential negative consequences of bias in clinical decision-making, it is incredibly important to not just examine the extent of bias in GAI responses but also to develop and implement strategies to reduce bias in GAI. Importantly, bias in decision-making by GAI models, including evidence of trauma-related diagnostic overshadowing, is likely driven by bias in data used to train GAI models [24,25,27].

Bias reduction strategies are especially critical in clinical contexts, where biased outputs could lead to misdiagnosis or ill-fitting treatment recommendations for trauma-exposed populations. GAI models are trained on biased data, including data that may reflect societal stereotypes or clinical misconceptions [24,25,27,29,44]. Approaches to bias reduction in GAI are particularly important and may include improved dataset curation, debiasing during model fine-tuning, prompt engineering techniques, human oversight methods, and post hoc bias correction mechanisms [29,44]. Educational strategies that help users recognize and mitigate model bias, such as bias demonstrations, feedback-based training, and consolidation of outputs across multiple models, have also shown promise [38,54,55]. Moreover, bias reduction must be considered across the full lifecycle of model development and deployment, including continuous auditing and version monitoring [55]. As GAI becomes increasingly integrated into mental health work, bias identification and mitigation are essential to ensure safe and effective outcomes. It is also particularly important to improve knowledge regarding GAI among professionals. Given the increasing integration of GAI in health care settings, it is imperative that health care professionals are equipped to understand, use, and evaluate these technologies as it relates to their practice. Improving health care professionals’ literacy around GAI can help mitigate risks associated with bias in clinical decision-making. Emerging work on AI literacy may provide a framework for evaluating and promoting essential knowledge for using GAI across domains [56]. As GAI becomes increasingly integrated into health care, it is essential that health care professionals are equipped not only to use these technologies but also to educate patients, laypeople, and other providers about their limitations.

There are significant ethical concerns related to the integration of GAI into mental health care. Although GAI systems offer potential benefits, such as enhancing accurate clinical decision-making, these systems also pose risks [28,29]. GAI models can inadvertently reinforce harmful stereotypes or produce misleading recommendations due to inherent biases in their training data, particularly when applied to trauma-exposed, minoritized, or otherwise vulnerable populations. Given that GAI lacks human judgment and contextual understanding, relying too heavily on its outputs without clinical oversight could lead to inappropriate treatment decisions or reduced patient trust [57]. Furthermore, overreliance on GAI for clinical decision-making may erode providers’ abilities to engage in these tasks. As these tools become more integrated into mental health settings, ethical guidelines must be developed to ensure responsible deployment, including greater involvement of providers in model training and testing, transparency in how models are trained, clear delineation of clinical responsibility, and safeguards to protect clinical populations [29,55-57]. Furthermore, as GAI continues to be scaled into health care technologies, it is increasingly important to embed intentional design choices in these technologies to minimize the risk of inaccurate, biased, or otherwise harmful decisions [57].

A few limitations should be noted. Primarily, research on trauma-related diagnostic overshadowing to date has been experimental. The vignettes in our study were brief and may not provide as much information as would be available in real-world clinical care settings. The specific prompting strategies used in this study may not reflect how clinicians interact with GAI tools in real-world practice. Clinicians are unlikely to use fixed Likert-based or forced-choice questions when using these tools and the context in which prompts are given can vary considerably. More research is needed to understand how MHPs may prompt GAI in real-world contexts [57]. Although standardized prompts were used in this study, slight variations on these prompts may result in different responses, including responses that may contain hallucinations or inaccurate responses. To match the paradigm for MHPs, prompts were not counterbalanced across trials, which is a limitation given the sensitivity of GAI models to prompt order and phrasing. While this fixed prompt order was necessary to mirror the methodology for MHPs, future work should explore how alternative or counterbalanced prompting strategies influence responses as it relates to clinical decision-making with trauma-exposed populations.

Furthermore, past work has shown that previous versions of specific GAI models can struggle to provide appropriate and safe guidance in response to complex clinical information [19,58]. As such, more research incorporating different streams of information, clinical presentations, and experimental methodologies is necessary to understand how GAI models can best support clinical decision-making with trauma-exposed populations. Moreover, more research is needed to understand how this bias contributes to decision-making in real-world contexts. In doing so, it may be important to understand if and how MHPs may be currently using GAI to support clinical decision-making with trauma-exposed populations. Only a few diagnostic presentations were considered for this research, and GAI models as well as MHPs may demonstrate differences in trauma-related diagnostic overshadowing in response to different symptom presentations, as shown in this work. This research also used a structured experimental paradigm, limiting our understanding of how GAI models may respond to less-structured prompts (eg, prompts in which diagnoses and treatment options are not provided). Ultimately, GAI models are often updated quickly, resulting in differences in their capabilities and outputs. As a result, findings from this study may not generalize to future versions of these models or to different applications of GAI within mental health care. More work may be needed to evaluate this bias in future versions of GAI models.

Despite these limitations, our study is the first to examine whether GAI models demonstrate trauma-related diagnostic overshadowing bias. Findings indicated that GAI models are not free of bias and the degree of bias is specific to the task and model. Notably, GAI models demonstrated less evidence of trauma-related diagnostic overshadowing than MHPs. GAI may be able to provide significant support to clinical decision-making in mental health care; however, there are important considerations for doing so. Future research should aim to further examine and develop strategies to address the presence of trauma-related diagnostic overshadowing among GAI models. In addition, research should aim to understand how best to use GAI to support MHPs to facilitate optimal clinical decision-making for trauma-exposed populations.

## Supplementary material

10.2196/80801 Multimedia Appendix 1 Experimental blocks containing vignettes and prompts.

10.2196/80801 Multimedia Appendix 2 Alignment of symptoms within vignettes with DSM-5-TR criteria.

10.2196/80801 Multimedia Appendix 3 Forced-choice selection of diagnosis and treatment options by generative artificial intelligence models.

## Abbreviations

CPT: cognitive processing therapy
ERP: exposure and response prevention
GAD: generalized anxiety disorder
GAI: generative artificial intelligence
MDD: major depressive disorder
MHP: mental health professional
MI: motivational interviewing
PTSD: posttraumatic stress disorder
SUD: substance use disorder
