# Supplementary material for: Comparing Generative Artificial Intelligence and Mental Health Professionals for Clinical Decision-Making With Trauma-Exposed Populations: Vignette-Based Experimental Study
Source: JMIR Ment Health. 2025 Oct 14;12:e80801. doi: 10.2196/80801 (PMC12527320; doi:10.2196/80801)
Supplement: Multimedia Appendix 1 [file mental-v12-e80801-s001.docx]

**Experimental Block #1:**

Please review the following case description:

“David, a 32-year-old white male, presents with longstanding difficulties with focus and anxiety. He has sought treatment, at the recommendation of his primary care physician. He has expressed concern with his declining performance at work and worsening relationship with his family and friends. He has never had prior mental health treatment but expresses openness to getting help. During your risk assessment, David denies experiencing suicidal ideation, non-suicidal self-injury, or significant substance abuse. During your screen for traumatic exposure, David endorses that he was sexually assaulted by a coworker on a business trip last year. David reports that the primary reason he has been struggling with work is that it takes hours to complete small tasks, both at home and at work. David reports excessive fear of accidentally sending inappropriate messages to his colleagues, friends, or family. He often rereads his emails and text messages numerous times, and he gets anxious if he does not check them before and after they are sent. David reports that he often gets distracted with thoughts of forgetting to lock his front door and car, and he spends hours each day checking them while at home. David also notes that, for the past year, it has been increasingly difficult for him to socialize with others, partly because he is afraid to leave the house in case he forgets to lock his door, causing someone to break in.”

Based on the case description above, how likely do you think David is to meet criteria for the following mental health diagnoses on a 7-point Likert scale (1 - Extremely Unlikely, 2, 3, 4 - Neither Likely or Unlikely, 5, 6, 7 - Extremely Likely, 9 - I Don’t Know Enough About This Diagnosis To Answer)? Your answer must be a whole number between 1 and 7. Please include just the diagnosis and your numeric response.

Adjustment Disorder

Generalized Anxiety Disorder

Major Depressive Disorder

Obsessive Compulsive Disorder

Post-Traumatic Stress Disorder

Substance Use Disorder

No Diagnosis

Of the following diagnoses, which do you feel is most likely to be the primary diagnosis that you would want to prioritize as an initial treatment target for David? Please select the primary diagnosis and include that below with no other information:

Adjustment Disorder

Generalized Anxiety Disorder

Major Depressive Disorder

Obsessive Compulsive Disorder

Post-Traumatic Stress Disorder

Substance Use Disorder

No Diagnosis

How appropriate do you feel the following treatment models are for addressing David’s symptom presentation on a 7-point Likert scale (1 - Extremely Inappropriate, 2, 3, 4 - Somewhat Appropriate, 5, 6, 7 - Extremely Appropriate; 9 - I Don't Know Enough To Answer)? Your answer must be a whole number between 1 and 7. Please include just the treatment and your numeric response.

Cognitive Processing Therapy

Dialectical Behavioral Therapy

Exposure and Response Prevention

Motivational Interviewing

Psychodynamic Psychotherapy

Other Treatment Model (If you selected 'other', please provide more information here on the model you chose in addition to your rating)

Of the following, which treatment model do you feel is the most clinically appropriate to address David’s symptom presentation outlined above? Please select the most clinically appropriate treatment model and include that below with no other information:

Cognitive Processing Therapy

Dialectical Behavioral Therapy

Exposure and Response Prevention

Motivational Interviewing

Psychodynamic Psychotherapy

Other Treatment Model (If you selected 'other', please provide more information here)

Please review the following case description:

“Laura, a 34-year-old white female, has recently begun receiving treatment at an outpatient clinic. She has sought treatment because of self-reported persistent difficulties with substance abuse involving alcohol. This is the first time she has sought treatment. During your risk assessment, she notes that she experiences mostly passive suicidal ideation, but she has no plan to harm herself. During your screen for traumatic exposure, Laura denies any exposure to trauma. Laura has noted that her drinking has led to declining performance at work. She reports consuming between 4-6 12oz. cans of beer each day for the past several years. When she is under the influence of alcohol, she can become irritable and she often initiates arguments with others. After these incidents, she reports feeling remorse and guilt. These events have put a strain on her relationships with her family and friends, and she reports partially seeking treatment to improve her relationships with them. Over the past few years, Laura has noted that she is often late for work after drinking the previous night. During your evaluation, Laura reports that she gets very upset easily and subsequently isolates herself.”

Based on the case description above, how likely do you think Laura is to meet criteria for the following mental health diagnoses on a 7-point Likert scale (1 - Extremely Unlikely, 2, 3, 4 - Neither Likely or Unlikely, 5, 6, 7 - Extremely Likely, 9 - I Don’t Know Enough About This Diagnosis To Answer)? Your answer must be a whole number between 1 and 7. Please include just the diagnosis and your numeric response.

Adjustment Disorder

Generalized Anxiety Disorder

Major Depressive Disorder

Obsessive Compulsive Disorder

Post-Traumatic Stress Disorder

Substance Use Disorder

No Diagnosis

Of the following diagnoses, which do you feel is most likely to be the primary diagnosis that you would want to prioritize as an initial treatment target for Laura? Please select the primary diagnosis and include that below with no other information:

Adjustment Disorder

Generalized Anxiety Disorder

Major Depressive Disorder

Obsessive Compulsive Disorder

Post-Traumatic Stress Disorder

Substance Use Disorder

No Diagnosis

How appropriate do you feel the following treatment models are for addressing Laura’s symptom presentation on a 7-point Likert scale (1 - Extremely Inappropriate, 2, 3, 4 - Somewhat Appropriate, 5, 6, 7 - Extremely Appropriate; 9 - I Don't Know Enough To Answer)? Your answer must be a whole number between 1 and 7. Please include just the treatment and your numeric response.

Cognitive Processing Therapy

Dialectical Behavioral Therapy

Exposure and Response Prevention

Motivational Interviewing

Psychodynamic Psychotherapy

Other Treatment Model (If you selected 'other', please provide more information here on the model you chose in addition to your rating)

Of the following, which treatment model do you feel is the most clinically appropriate to address Laura’s symptom presentation outlined above? Please select the most clinically appropriate treatment model and include that below with no other information:

Cognitive Processing Therapy

Dialectical Behavioral Therapy

Exposure and Response Prevention

Motivational Interviewing

Psychodynamic Psychotherapy

Other Treatment Model (If you selected 'other', please provide more information here)

**Experimental Block #2:**

Please review the following case description:

“David, a 32-year-old white male, presents with longstanding difficulties with focus and anxiety. He has sought treatment, at the recommendation of his primary care physician. He has expressed concern with his declining performance at work and worsening relationship with his family and friends. He has never had prior mental health treatment but expresses openness to getting help. During your risk assessment, David denies experiencing suicidal ideation, non-suicidal self-injury, or significant substance abuse. During your screen for traumatic exposure, David reports that he was in a serious car accident last year that left him with lingering physical injuries. David reports that the primary reason he has been struggling with work is that it takes hours to complete small tasks, both at home and at work. David reports excessive fear of accidentally sending inappropriate messages to his colleagues, friends, or family. He often rereads his emails and text messages numerous times, and he gets anxious if he does not check them before and after they are sent. David reports that he often gets distracted with thoughts of forgetting to lock his front door and car, and he spends hours each day checking them while at home. David also notes that, for the past year, it has been increasingly difficult for him to socialize with others, partly because he is afraid to leave the house in case he forgets to lock his door, causing someone to break in.”

Based on the case description above, how likely do you think David is to meet criteria for the following mental health diagnoses on a 7-point Likert scale (1 - Extremely Unlikely, 2, 3, 4 - Neither Likely or Unlikely, 5, 6, 7 - Extremely Likely, 9 - I Don’t Know Enough About This Diagnosis To Answer)? Your answer must be a whole number between 1 and 7. Please include just the diagnosis and your numeric response.

Adjustment Disorder

Generalized Anxiety Disorder

Major Depressive Disorder

Obsessive Compulsive Disorder

Post-Traumatic Stress Disorder

Substance Use Disorder

No Diagnosis

Of the following diagnoses, which do you feel is most likely to be the primary diagnosis that you would want to prioritize as an initial treatment target for David? Please select the primary diagnosis and include that below with no other information:

Adjustment Disorder

Generalized Anxiety Disorder

Major Depressive Disorder

Obsessive Compulsive Disorder

Post-Traumatic Stress Disorder

Substance Use Disorder

No Diagnosis

How appropriate do you feel the following treatment models are for addressing David’s symptom presentation on a 7-point Likert scale (1 - Extremely Inappropriate, 2, 3, 4 - Somewhat Appropriate, 5, 6, 7 - Extremely Appropriate; 9 - I Don't Know Enough To Answer)? Your answer must be a whole number between 1 and 7. Please include just the treatment and your numeric response.

Cognitive Processing Therapy

Dialectical Behavioral Therapy

Exposure and Response Prevention

Motivational Interviewing

Psychodynamic Psychotherapy

Other Treatment Model (If you selected 'other', please provide more information here on the model you chose in addition to your rating)

Of the following, which treatment model do you feel is the most clinically appropriate to address David’s symptom presentation outlined above? Please select the most clinically appropriate treatment model and include that below with no other information:

Cognitive Processing Therapy

Dialectical Behavioral Therapy

Exposure and Response Prevention

Motivational Interviewing

Psychodynamic Psychotherapy

Other Treatment Model (If you selected 'other', please provide more information here)

Please review the following case description:

“Laura, a 34-year-old white female, has recently begun receiving treatment at an outpatient clinic. She has sought treatment because of self-reported persistent difficulties with substance abuse involving alcohol. This is the first time she has sought treatment. During your risk assessment, she notes that she experiences mostly passive suicidal ideation, but she has no plan to harm herself. During your screen for traumatic exposure, Laura denies any exposure to trauma. Laura has noted that her drinking has led to declining performance at work. She reports consuming between 4-6 12oz. cans of beer each day for the past several years. When she is under the influence of alcohol, she can become irritable and she often initiates arguments with others. After these incidents, she reports feeling remorse and guilt. These events have put a strain on her relationships with her family and friends, and she reports partially seeking treatment to improve her relationships with them. Over the past few years, Laura has noted that she is often late for work after drinking the previous night. During your evaluation, Laura reports that she gets very upset easily and subsequently isolates herself.”

Based on the case description above, how likely do you think Laura is to meet criteria for the following mental health diagnoses on a 7-point Likert scale (1 - Extremely Unlikely, 2, 3, 4 - Neither Likely or Unlikely, 5, 6, 7 - Extremely Likely, 9 - I Don’t Know Enough About This Diagnosis To Answer)? Your answer must be a whole number between 1 and 7. Please include just the diagnosis and your numeric response.

Adjustment Disorder

Generalized Anxiety Disorder

Major Depressive Disorder

Obsessive Compulsive Disorder

Post-Traumatic Stress Disorder

Substance Use Disorder

No Diagnosis

Of the following diagnoses, which do you feel is most likely to be the primary diagnosis that you would want to prioritize as an initial treatment target for Laura? Please select the primary diagnosis and include that below with no other information:

Adjustment Disorder

Generalized Anxiety Disorder

Major Depressive Disorder

Obsessive Compulsive Disorder

Post-Traumatic Stress Disorder

Substance Use Disorder

No Diagnosis

How appropriate do you feel the following treatment models are for addressing Laura’s symptom presentation on a 7-point Likert scale (1 - Extremely Inappropriate, 2, 3, 4 - Somewhat Appropriate, 5, 6, 7 - Extremely Appropriate; 9 - I Don't Know Enough To Answer)? Your answer must be a whole number between 1 and 7. Please include just the treatment and your numeric response.

Cognitive Processing Therapy

Dialectical Behavioral Therapy

Exposure and Response Prevention

Motivational Interviewing

Psychodynamic Psychotherapy

Other Treatment Model (If you selected 'other', please provide more information here on the model you chose in addition to your rating)

Of the following, which treatment model do you feel is the most clinically appropriate to address Laura’s symptom presentation outlined above? Please select the most clinically appropriate treatment model and include that below with no other information:

Cognitive Processing Therapy

Dialectical Behavioral Therapy

Exposure and Response Prevention

Motivational Interviewing

Psychodynamic Psychotherapy

Other Treatment Model (If you selected 'other', please provide more information here)

**Experimental Block #3:**

Please review the following case description:

“David, a 32-year-old white male, presents with longstanding difficulties with focus and anxiety. He has sought treatment, at the recommendation of his primary care physician. He has expressed concern with his declining performance at work and worsening relationship with his family and friends. He has never had prior mental health treatment but expresses openness to getting help. During your risk assessment, David denies experiencing suicidal ideation, non-suicidal self-injury, or significant substance abuse. During your screen for traumatic exposure, David denies any exposure to trauma. David reports that the primary reason he has been struggling with work is that it takes hours to complete small tasks, both at home and at work. David reports excessive fear of accidentally sending inappropriate messages to his colleagues, friends, or family. He often rereads his emails and text messages numerous times, and he gets anxious if he does not check them before and after they are sent. David reports that he often gets distracted with thoughts of forgetting to lock his front door and car, and he spends hours each day checking them while at home. David also notes that, for the past year, it has been increasingly difficult for him to socialize with others, partly because he is afraid to leave the house in case he forgets to lock his door, causing someone to break in.”

Based on the case description above, how likely do you think David is to meet criteria for the following mental health diagnoses on a 7-point Likert scale (1 - Extremely Unlikely, 2, 3, 4 - Neither Likely or Unlikely, 5, 6, 7 - Extremely Likely, 9 - I Don’t Know Enough About This Diagnosis To Answer)? Your answer must be a whole number between 1 and 7. Please include just the diagnosis and your numeric response.

Adjustment Disorder

Generalized Anxiety Disorder

Major Depressive Disorder

Obsessive Compulsive Disorder

Post-Traumatic Stress Disorder

Substance Use Disorder

No Diagnosis

Of the following diagnoses, which do you feel is most likely to be the primary diagnosis that you would want to prioritize as an initial treatment target for David? Please select the primary diagnosis and include that below with no other information:

Adjustment Disorder

Generalized Anxiety Disorder

Major Depressive Disorder

Obsessive Compulsive Disorder

Post-Traumatic Stress Disorder

Substance Use Disorder

No Diagnosis

How appropriate do you feel the following treatment models are for addressing David’s symptom presentation on a 7-point Likert scale (1 - Extremely Inappropriate, 2, 3, 4 - Somewhat Appropriate, 5, 6, 7 - Extremely Appropriate; 9 - I Don't Know Enough To Answer)? Your answer must be a whole number between 1 and 7. Please include just the treatment and your numeric response.

Cognitive Processing Therapy

Dialectical Behavioral Therapy

Exposure and Response Prevention

Motivational Interviewing

Psychodynamic Psychotherapy

Other Treatment Model (If you selected 'other', please provide more information here on the model you chose in addition to your rating)

Of the following, which treatment model do you feel is the most clinically appropriate to address David’s symptom presentation outlined above? Please select the most clinically appropriate treatment model and include that below with no other information:

Cognitive Processing Therapy

Dialectical Behavioral Therapy

Exposure and Response Prevention

Motivational Interviewing

Psychodynamic Psychotherapy

Other Treatment Model (If you selected 'other', please provide more information here)

Please review the following case description:

“Laura, a 34-year-old white female, has recently begun receiving treatment at an outpatient clinic. She has sought treatment because of self-reported persistent difficulties with substance abuse involving alcohol. This is the first time she has sought treatment. During your risk assessment, she notes that she experiences mostly passive suicidal ideation, but she has no plan to harm herself. During your screen for traumatic exposure, Laura endorses that she was sexually assaulted by a coworker on a business trip last year. Laura has noted that her drinking has led to declining performance at work. She reports consuming between 4-6 12oz. cans of beer each day for the past several years. When she is under the influence of alcohol, she can become irritable and she often initiates arguments with others. After these incidents, she reports feeling remorse and guilt. These events have put a strain on her relationships with her family and friends, and she reports partially seeking treatment to improve her relationships with them. Over the past few years, Laura has noted that she is often late for work after drinking the previous night. During your evaluation, Laura reports that she gets very upset easily and subsequently isolates herself.”

Based on the case description above, how likely do you think Laura is to meet criteria for the following mental health diagnoses on a 7-point Likert scale (1 - Extremely Unlikely, 2, 3, 4 - Neither Likely or Unlikely, 5, 6, 7 - Extremely Likely, 9 - I Don’t Know Enough About This Diagnosis To Answer)? Your answer must be a whole number between 1 and 7. Please include just the diagnosis and your numeric response.

Adjustment Disorder

Generalized Anxiety Disorder

Major Depressive Disorder

Obsessive Compulsive Disorder

Post-Traumatic Stress Disorder

Substance Use Disorder

No Diagnosis

Of the following diagnoses, which do you feel is most likely to be the primary diagnosis that you would want to prioritize as an initial treatment target for Laura? Please select the primary diagnosis and include that below with no other information:

Adjustment Disorder

Generalized Anxiety Disorder

Major Depressive Disorder

Obsessive Compulsive Disorder

Post-Traumatic Stress Disorder

Substance Use Disorder

No Diagnosis

How appropriate do you feel the following treatment models are for addressing Laura’s symptom presentation on a 7-point Likert scale (1 - Extremely Inappropriate, 2, 3, 4 - Somewhat Appropriate, 5, 6, 7 - Extremely Appropriate; 9 - I Don't Know Enough To Answer)? Your answer must be a whole number between 1 and 7. Please include just the treatment and your numeric response.

Cognitive Processing Therapy

Dialectical Behavioral Therapy

Exposure and Response Prevention

Motivational Interviewing

Psychodynamic Psychotherapy

Other Treatment Model (If you selected 'other', please provide more information here on the model you chose in addition to your rating)

Of the following, which treatment model do you feel is the most clinically appropriate to address Laura’s symptom presentation outlined above? Please select the most clinically appropriate treatment model and include that below with no other information:

Cognitive Processing Therapy

Dialectical Behavioral Therapy

Exposure and Response Prevention

Motivational Interviewing

Psychodynamic Psychotherapy

Other Treatment Model (If you selected 'other', please provide more information here)

**Experimental Block #4:**

Please review the following case description:

“David, a 32-year-old white male, presents with longstanding difficulties with focus and anxiety. He has sought treatment, at the recommendation of his primary care physician. He has expressed concern with his declining performance at work and worsening relationship with his family and friends. He has never had prior mental health treatment but expresses openness to getting help. During your risk assessment, David denies experiencing suicidal ideation, non-suicidal self-injury, or significant substance abuse. During your screen for traumatic exposure, David denies any exposure to trauma. David reports that the primary reason he has been struggling with work is that it takes hours to complete small tasks, both at home and at work. David reports excessive fear of accidentally sending inappropriate messages to his colleagues, friends, or family. He often rereads his emails and text messages numerous times, and he gets anxious if he does not check them before and after they are sent. David reports that he often gets distracted with thoughts of forgetting to lock his front door and car, and he spends hours each day checking them while at home. David also notes that, for the past year, it has been increasingly difficult for him to socialize with others, partly because he is afraid to leave the house in case he forgets to lock his door, causing someone to break in.”

Based on the case description above, how likely do you think David is to meet criteria for the following mental health diagnoses on a 7-point Likert scale (1 - Extremely Unlikely, 2, 3, 4 - Neither Likely or Unlikely, 5, 6, 7 - Extremely Likely, 9 - I Don’t Know Enough About This Diagnosis To Answer)? Your answer must be a whole number between 1 and 7. Please include just the diagnosis and your numeric response.

Adjustment Disorder

Generalized Anxiety Disorder

Major Depressive Disorder

Obsessive Compulsive Disorder

Post-Traumatic Stress Disorder

Substance Use Disorder

No Diagnosis

Of the following diagnoses, which do you feel is most likely to be the primary diagnosis that you would want to prioritize as an initial treatment target for David? Please select the primary diagnosis and include that below with no other information:

Adjustment Disorder

Generalized Anxiety Disorder

Major Depressive Disorder

Obsessive Compulsive Disorder

Post-Traumatic Stress Disorder

Substance Use Disorder

No Diagnosis

How appropriate do you feel the following treatment models are for addressing David’s symptom presentation on a 7-point Likert scale (1 - Extremely Inappropriate, 2, 3, 4 - Somewhat Appropriate, 5, 6, 7 - Extremely Appropriate; 9 - I Don't Know Enough To Answer)? Your answer must be a whole number between 1 and 7. Please include just the treatment and your numeric response.

Cognitive Processing Therapy

Dialectical Behavioral Therapy

Exposure and Response Prevention

Motivational Interviewing

Psychodynamic Psychotherapy

Other Treatment Model (If you selected 'other', please provide more information here on the model you chose in addition to your rating)

Of the following, which treatment model do you feel is the most clinically appropriate to address David’s symptom presentation outlined above? Please select the most clinically appropriate treatment model and include that below with no other information:

Cognitive Processing Therapy

Dialectical Behavioral Therapy

Exposure and Response Prevention

Motivational Interviewing

Psychodynamic Psychotherapy

Other Treatment Model (If you selected 'other', please provide more information here)

Please review the following case description:

“Laura, a 34-year-old white female, has recently begun receiving treatment at an outpatient clinic. She has sought treatment because of self-reported persistent difficulties with substance abuse involving alcohol. This is the first time she has sought treatment. During your risk assessment, she notes that she experiences mostly passive suicidal ideation, but she has no plan to harm herself. During your screen for traumatic exposure, Laura reports that she was in a serious car accident last year that left her with lingering physical injuries. Laura has noted that her drinking has led to declining performance at work. She reports consuming between 4-6 12oz. cans of beer each day for the past several years. When she is under the influence of alcohol, she can become irritable and she often initiates arguments with others. After these incidents, she reports feeling remorse and guilt. These events have put a strain on her relationships with her family and friends, and she reports partially seeking treatment to improve her relationships with them. Over the past few years, Laura has noted that she is often late for work after drinking the previous night. During your evaluation, Laura reports that she gets very upset easily and subsequently isolates herself.”

Based on the case description above, how likely do you think Laura is to meet criteria for the following mental health diagnoses on a 7-point Likert scale (1 - Extremely Unlikely, 2, 3, 4 - Neither Likely or Unlikely, 5, 6, 7 - Extremely Likely, 9 - I Don’t Know Enough About This Diagnosis To Answer)? Your answer must be a whole number between 1 and 7. Please include just the diagnosis and your numeric response.

Adjustment Disorder

Generalized Anxiety Disorder

Major Depressive Disorder

Obsessive Compulsive Disorder

Post-Traumatic Stress Disorder

Substance Use Disorder

No Diagnosis

Of the following diagnoses, which do you feel is most likely to be the primary diagnosis that you would want to prioritize as an initial treatment target for Laura? Please select the primary diagnosis and include that below with no other information:

Adjustment Disorder

Generalized Anxiety Disorder

Major Depressive Disorder

Obsessive Compulsive Disorder

Post-Traumatic Stress Disorder

Substance Use Disorder

No Diagnosis

How appropriate do you feel the following treatment models are for addressing Laura’s symptom presentation on a 7-point Likert scale (1 - Extremely Inappropriate, 2, 3, 4 - Somewhat Appropriate, 5, 6, 7 - Extremely Appropriate; 9 - I Don't Know Enough To Answer)? Your answer must be a whole number between 1 and 7. Please include just the treatment and your numeric response.

Cognitive Processing Therapy

Dialectical Behavioral Therapy

Exposure and Response Prevention

Motivational Interviewing

Psychodynamic Psychotherapy

Other Treatment Model (If you selected 'other', please provide more information here on the model you chose in addition to your rating)

Of the following, which treatment model do you feel is the most clinically appropriate to address Laura’s symptom presentation outlined above? Please select the most clinically appropriate treatment model and include that below with no other information:

Cognitive Processing Therapy

Dialectical Behavioral Therapy

Exposure and Response Prevention

Motivational Interviewing

Psychodynamic Psychotherapy

Other Treatment Model (If you selected 'other', please provide more information here)

**Experimental Block #5:**

Please review the following case description:

“Laura, a 32-year-old white female, presents with longstanding difficulties with focus and anxiety. She has sought treatment, at the recommendation of her primary care physician. She has expressed concern with her declining performance at work and worsening relationship with her family and friends. She has never had prior mental health treatment but expresses openness to getting help. During your risk assessment, Laura denies experiencing suicidal ideation, non-suicidal self-injury, or significant substance abuse. During your screen for traumatic exposure, Laura endorses that she was sexually assaulted by a coworker on a business trip last year. Laura reports that the primary reason she has been struggling with work is that it takes hours to complete small tasks, both at home and at work. Laura reports excessive fear of accidentally sending inappropriate messages to her colleagues, friends, or family. She often rereads her emails and text messages numerous times, and she gets anxious if she does not check them before and after they are sent. Laura reports that she often gets distracted with thoughts of forgetting to lock her front door and car, and she spends hours each day checking them while at home. Laura also notes that, for the past year, it has been increasingly difficult for her to socialize with others, partly because she is afraid to leave the house in case she forgets to lock her door, causing someone to break in.”

Based on the case description above, how likely do you think Laura is to meet criteria for the following mental health diagnoses on a 7-point Likert scale (1 - Extremely Unlikely, 2, 3, 4 - Neither Likely or Unlikely, 5, 6, 7 - Extremely Likely, 9 - I Don’t Know Enough About This Diagnosis To Answer)? Your answer must be a whole number between 1 and 7. Please include just the diagnosis and your numeric response.

Adjustment Disorder

Generalized Anxiety Disorder

Major Depressive Disorder

Obsessive Compulsive Disorder

Post-Traumatic Stress Disorder

Substance Use Disorder

No Diagnosis

Of the following diagnoses, which do you feel is most likely to be the primary diagnosis that you would want to prioritize as an initial treatment target for Laura? Please select the primary diagnosis and include that below with no other information:

Adjustment Disorder

Generalized Anxiety Disorder

Major Depressive Disorder

Obsessive Compulsive Disorder

Post-Traumatic Stress Disorder

Substance Use Disorder

No Diagnosis

How appropriate do you feel the following treatment models are for addressing Laura’s symptom presentation on a 7-point Likert scale (1 - Extremely Inappropriate, 2, 3, 4 - Somewhat Appropriate, 5, 6, 7 - Extremely Appropriate; 9 - I Don't Know Enough To Answer)? Your answer must be a whole number between 1 and 7. Please include just the treatment and your numeric response.

Cognitive Processing Therapy

Dialectical Behavioral Therapy

Exposure and Response Prevention

Motivational Interviewing

Psychodynamic Psychotherapy

Other Treatment Model (If you selected 'other', please provide more information here on the model you chose in addition to your rating)

Of the following, which treatment model do you feel is the most clinically appropriate to address Laura’s symptom presentation outlined above? Please select the most clinically appropriate treatment model and include that below with no other information:

Cognitive Processing Therapy

Dialectical Behavioral Therapy

Exposure and Response Prevention

Motivational Interviewing

Psychodynamic Psychotherapy

Other Treatment Model (If you selected 'other', please provide more information here)

Please review the following case description:

“David, a 34-year-old white male, has recently begun receiving treatment at an outpatient clinic. He has sought treatment because of self-reported persistent difficulties with substance abuse involving alcohol. This is the first time he has sought treatment. During your risk assessment, he notes that he experiences mostly passive suicidal ideation, but he has no plan to harm himself. During your screen for traumatic exposure, David denies any exposure to trauma. David has noted that his drinking has led to declining performance at work. He reports consuming between 6-8 12oz. cans of beer each day for the past several years. When he is under the influence of alcohol, he can become irritable and he often initiates arguments with others. After these incidents, he reports feeling remorse and guilt. These events have put a strain on his relationships with his family and friends, and he reports partially seeking treatment to improve his relationships with them. Over the past few years, David has noted that he is often late for work after drinking the previous night. During your evaluation, David reports that he gets very upset easily and subsequently isolates himself.”

Based on the case description above, how likely do you think David is to meet criteria for the following mental health diagnoses on a 7-point Likert scale (1 - Extremely Unlikely, 2, 3, 4 - Neither Likely or Unlikely, 5, 6, 7 - Extremely Likely, 9 - I Don’t Know Enough About This Diagnosis To Answer)? Your answer must be a whole number between 1 and 7. Please include just the diagnosis and your numeric response.

Adjustment Disorder

Generalized Anxiety Disorder

Major Depressive Disorder

Obsessive Compulsive Disorder

Post-Traumatic Stress Disorder

Substance Use Disorder

No Diagnosis

Of the following diagnoses, which do you feel is most likely to be the primary diagnosis that you would want to prioritize as an initial treatment target for David? Please select the primary diagnosis and include that below with no other information:

Adjustment Disorder

Generalized Anxiety Disorder

Major Depressive Disorder

Obsessive Compulsive Disorder

Post-Traumatic Stress Disorder

Substance Use Disorder

No Diagnosis

How appropriate do you feel the following treatment models are for addressing David’s symptom presentation on a 7-point Likert scale (1 - Extremely Inappropriate, 2, 3, 4 - Somewhat Appropriate, 5, 6, 7 - Extremely Appropriate; 9 - I Don't Know Enough To Answer)? Your answer must be a whole number between 1 and 7. Please include just the treatment and your numeric response.

Cognitive Processing Therapy

Dialectical Behavioral Therapy

Exposure and Response Prevention

Motivational Interviewing

Psychodynamic Psychotherapy

Other Treatment Model (If you selected 'other', please provide more information here on the model you chose in addition to your rating)

Of the following, which treatment model do you feel is the most clinically appropriate to address David’s symptom presentation outlined above? Please select the most clinically appropriate treatment model and include that below with no other information:

Cognitive Processing Therapy

Dialectical Behavioral Therapy

Exposure and Response Prevention

Motivational Interviewing

Psychodynamic Psychotherapy

Other Treatment Model (If you selected 'other', please provide more information here)

**Experimental Block #6:**

Please review the following case description:

“Laura, a 32-year-old white female, presents with longstanding difficulties with focus and anxiety. She has sought treatment, at the recommendation of her primary care physician. She has expressed concern with her declining performance at work and worsening relationship with her family and friends. She has never had prior mental health treatment but expresses openness to getting help. During your risk assessment, Laura denies experiencing suicidal ideation, non-suicidal self-injury, or significant substance abuse. During your screen for traumatic exposure, Laura endorses that she was in a major car accident last year that left her with lingering physical injuries. Laura reports that the primary reason she has been struggling with work is that it takes hours to complete small tasks, both at home and at work. Laura reports excessive fear of accidentally sending inappropriate messages to her colleagues, friends, or family. She often rereads her emails and text messages numerous times, and she gets anxious if she does not check them before and after they are sent. Laura reports that she often gets distracted with thoughts of forgetting to lock her front door and car, and she spends hours each day checking them while at home. Laura also notes that, for the past year, it has been increasingly difficult for her to socialize with others, partly because she is afraid to leave the house in case she forgets to lock her door, causing someone to break in.”

Based on the case description above, how likely do you think Laura is to meet criteria for the following mental health diagnoses on a 7-point Likert scale (1 - Extremely Unlikely, 2, 3, 4 - Neither Likely or Unlikely, 5, 6, 7 - Extremely Likely, 9 - I Don’t Know Enough About This Diagnosis To Answer)? Your answer must be a whole number between 1 and 7. Please include just the diagnosis and your numeric response.

Adjustment Disorder

Generalized Anxiety Disorder

Major Depressive Disorder

Obsessive Compulsive Disorder

Post-Traumatic Stress Disorder

Substance Use Disorder

No Diagnosis

Of the following diagnoses, which do you feel is most likely to be the primary diagnosis that you would want to prioritize as an initial treatment target for Laura? Please select the primary diagnosis and include that below with no other information:

Adjustment Disorder

Generalized Anxiety Disorder

Major Depressive Disorder

Obsessive Compulsive Disorder

Post-Traumatic Stress Disorder

Substance Use Disorder

No Diagnosis

How appropriate do you feel the following treatment models are for addressing Laura’s symptom presentation on a 7-point Likert scale (1 - Extremely Inappropriate, 2, 3, 4 - Somewhat Appropriate, 5, 6, 7 - Extremely Appropriate; 9 - I Don't Know Enough To Answer)? Your answer must be a whole number between 1 and 7. Please include just the treatment and your numeric response.

Cognitive Processing Therapy

Dialectical Behavioral Therapy

Exposure and Response Prevention

Motivational Interviewing

Psychodynamic Psychotherapy

Other Treatment Model (If you selected 'other', please provide more information here on the model you chose in addition to your rating)

Of the following, which treatment model do you feel is the most clinically appropriate to address Laura’s symptom presentation outlined above? Please select the most clinically appropriate treatment model and include that below with no other information:

Cognitive Processing Therapy

Dialectical Behavioral Therapy

Exposure and Response Prevention

Motivational Interviewing

Psychodynamic Psychotherapy

Other Treatment Model (If you selected 'other', please provide more information here)

Please review the following case description:

“David, a 34-year-old white male, has recently begun receiving treatment at an outpatient clinic. He has sought treatment because of self-reported persistent difficulties with substance abuse involving alcohol. This is the first time he has sought treatment. During your risk assessment, he notes that he experiences mostly passive suicidal ideation, but he has no plan to harm himself. During your screen for traumatic exposure, David denies any exposure to trauma. David has noted that his drinking has led to declining performance at work. He reports consuming between 6-8 12oz. cans of beer each day for the past several years. When he is under the influence of alcohol, he can become irritable and he often initiates arguments with others. After these incidents, he reports feeling remorse and guilt. These events have put a strain on his relationships with his family and friends, and he reports partially seeking treatment to improve his relationships with them. Over the past few years, David has noted that he is often late for work after drinking the previous night. During your evaluation, David reports that he gets very upset easily and subsequently isolates himself.”

Based on the case description above, how likely do you think David is to meet criteria for the following mental health diagnoses on a 7-point Likert scale (1 - Extremely Unlikely, 2, 3, 4 - Neither Likely or Unlikely, 5, 6, 7 - Extremely Likely, 9 - I Don’t Know Enough About This Diagnosis To Answer)? Your answer must be a whole number between 1 and 7. Please include just the diagnosis and your numeric response.

Adjustment Disorder

Generalized Anxiety Disorder

Major Depressive Disorder

Obsessive Compulsive Disorder

Post-Traumatic Stress Disorder

Substance Use Disorder

No Diagnosis

Of the following diagnoses, which do you feel is most likely to be the primary diagnosis that you would want to prioritize as an initial treatment target for David? Please select the primary diagnosis and include that below with no other information:

Adjustment Disorder

Generalized Anxiety Disorder

Major Depressive Disorder

Obsessive Compulsive Disorder

Post-Traumatic Stress Disorder

Substance Use Disorder

No Diagnosis

How appropriate do you feel the following treatment models are for addressing David’s symptom presentation on a 7-point Likert scale (1 - Extremely Inappropriate, 2, 3, 4 - Somewhat Appropriate, 5, 6, 7 - Extremely Appropriate; 9 - I Don't Know Enough To Answer)? Your answer must be a whole number between 1 and 7. Please include just the treatment and your numeric response.

Cognitive Processing Therapy

Dialectical Behavioral Therapy

Exposure and Response Prevention

Motivational Interviewing

Psychodynamic Psychotherapy

Other Treatment Model (If you selected 'other', please provide more information here on the model you chose in addition to your rating)

Of the following, which treatment model do you feel is the most clinically appropriate to address David’s symptom presentation outlined above? Please select the most clinically appropriate treatment model and include that below with no other information:

Cognitive Processing Therapy

Dialectical Behavioral Therapy

Exposure and Response Prevention

Motivational Interviewing

Psychodynamic Psychotherapy

Other Treatment Model (If you selected 'other', please provide more information here)

**Experimental Block #7:**

Please review the following case description:

“Laura, a 32-year-old white female, presents with longstanding difficulties with focus and anxiety. She has sought treatment, at the recommendation of her primary care physician. She has expressed concern with her declining performance at work and worsening relationship with her family and friends. She has never had prior mental health treatment but expresses openness to getting help. During your risk assessment, Laura denies experiencing suicidal ideation, non-suicidal self-injury, or significant substance abuse. During your screen for traumatic exposure, Laura denies any exposure to trauma. Laura reports that the primary reason she has been struggling with work is that it takes hours to complete small tasks, both at home and at work. Laura reports excessive fear of accidentally sending inappropriate messages to her colleagues, friends, or family. She often rereads her emails and text messages numerous times, and she gets anxious if she does not check them before and after they are sent. Laura reports that she often gets distracted with thoughts of forgetting to lock her front door and car, and she spends hours each day checking them while at home. Laura also notes that, for the past year, it has been increasingly difficult for her to socialize with others, partly because she is afraid to leave the house in case she forgets to lock her door, causing someone to break in.”

Based on the case description above, how likely do you think Laura is to meet criteria for the following mental health diagnoses on a 7-point Likert scale (1 - Extremely Unlikely, 2, 3, 4 - Neither Likely or Unlikely, 5, 6, 7 - Extremely Likely, 9 - I Don’t Know Enough About This Diagnosis To Answer)? Your answer must be a whole number between 1 and 7. Please include just the diagnosis and your numeric response.

Adjustment Disorder

Generalized Anxiety Disorder

Major Depressive Disorder

Obsessive Compulsive Disorder

Post-Traumatic Stress Disorder

Substance Use Disorder

No Diagnosis

Of the following diagnoses, which do you feel is most likely to be the primary diagnosis that you would want to prioritize as an initial treatment target for Laura? Please select the primary diagnosis and include that below with no other information:

Adjustment Disorder

Generalized Anxiety Disorder

Major Depressive Disorder

Obsessive Compulsive Disorder

Post-Traumatic Stress Disorder

Substance Use Disorder

No Diagnosis

How appropriate do you feel the following treatment models are for addressing Laura’s symptom presentation on a 7-point Likert scale (1 - Extremely Inappropriate, 2, 3, 4 - Somewhat Appropriate, 5, 6, 7 - Extremely Appropriate; 9 - I Don't Know Enough To Answer)? Your answer must be a whole number between 1 and 7. Please include just the treatment and your numeric response.

Cognitive Processing Therapy

Dialectical Behavioral Therapy

Exposure and Response Prevention

Motivational Interviewing

Psychodynamic Psychotherapy

Other Treatment Model (If you selected 'other', please provide more information here on the model you chose in addition to your rating)

Of the following, which treatment model do you feel is the most clinically appropriate to address Laura’s symptom presentation outlined above? Please select the most clinically appropriate treatment model and include that below with no other information:

Cognitive Processing Therapy

Dialectical Behavioral Therapy

Exposure and Response Prevention

Motivational Interviewing

Psychodynamic Psychotherapy

Other Treatment Model (If you selected 'other', please provide more information here)

Please review the following case description:

“David, a 34-year-old white male, has recently begun receiving treatment at an outpatient clinic. He has sought treatment because of self-reported persistent difficulties with substance abuse involving alcohol. This is the first time he has sought treatment. During your risk assessment, he notes that he experiences mostly passive suicidal ideation, but he has no plan to harm himself. During your screen for traumatic exposure, David endorses that he was sexually assaulted by a coworker on a business trip last year. David has noted that his drinking has led to declining performance at work. He reports consuming between 6-8 12oz. cans of beer each day for the past several years. When he is under the influence of alcohol, he can become irritable and he often initiates arguments with others. After these incidents, he reports feeling remorse and guilt. These events have put a strain on his relationships with his family and friends, and he reports partially seeking treatment to improve his relationships with them. Over the past few years, David has noted that he is often late for work after drinking the previous night. During your evaluation, David reports that he gets very upset easily and subsequently isolates himself.”

Based on the case description above, how likely do you think David is to meet criteria for the following mental health diagnoses on a 7-point Likert scale (1 - Extremely Unlikely, 2, 3, 4 - Neither Likely or Unlikely, 5, 6, 7 - Extremely Likely, 9 - I Don’t Know Enough About This Diagnosis To Answer)? Your answer must be a whole number between 1 and 7. Please include just the diagnosis and your numeric response.

Adjustment Disorder

Generalized Anxiety Disorder

Major Depressive Disorder

Obsessive Compulsive Disorder

Post-Traumatic Stress Disorder

Substance Use Disorder

No Diagnosis

Of the following diagnoses, which do you feel is most likely to be the primary diagnosis that you would want to prioritize as an initial treatment target for David? Please select the primary diagnosis and include that below with no other information:

Adjustment Disorder

Generalized Anxiety Disorder

Major Depressive Disorder

Obsessive Compulsive Disorder

Post-Traumatic Stress Disorder

Substance Use Disorder

No Diagnosis

How appropriate do you feel the following treatment models are for addressing David’s symptom presentation on a 7-point Likert scale (1 - Extremely Inappropriate, 2, 3, 4 - Somewhat Appropriate, 5, 6, 7 - Extremely Appropriate; 9 - I Don't Know Enough To Answer)? Your answer must be a whole number between 1 and 7. Please include just the treatment and your numeric response.

Cognitive Processing Therapy

Dialectical Behavioral Therapy

Exposure and Response Prevention

Motivational Interviewing

Psychodynamic Psychotherapy

Other Treatment Model (If you selected 'other', please provide more information here on the model you chose in addition to your rating)

Of the following, which treatment model do you feel is the most clinically appropriate to address David’s symptom presentation outlined above? Please select the most clinically appropriate treatment model and include that below with no other information:

Cognitive Processing Therapy

Dialectical Behavioral Therapy

Exposure and Response Prevention

Motivational Interviewing

Psychodynamic Psychotherapy

Other Treatment Model (If you selected 'other', please provide more information here)

**Experimental Block #8:**

Please review the following case description:

“Laura, a 32-year-old white female, presents with longstanding difficulties with focus and anxiety. She has sought treatment, at the recommendation of her primary care physician. She has expressed concern with her declining performance at work and worsening relationship with her family and friends. She has never had prior mental health treatment but expresses openness to getting help. During your risk assessment, Laura denies experiencing suicidal ideation, non-suicidal self-injury, or significant substance abuse. During your screen for traumatic exposure, Laura denies any exposure to trauma. Laura reports that the primary reason she has been struggling with work is that it takes hours to complete small tasks, both at home and at work. Laura reports excessive fear of accidentally sending inappropriate messages to her colleagues, friends, or family. She often rereads her emails and text messages numerous times, and she gets anxious if she does not check them before and after they are sent. Laura reports that she often gets distracted with thoughts of forgetting to lock her front door and car, and she spends hours each day checking them while at home. Laura also notes that, for the past year, it has been increasingly difficult for her to socialize with others, partly because she is afraid to leave the house in case she forgets to lock her door, causing someone to break in.”

Based on the case description above, how likely do you think Laura is to meet criteria for the following mental health diagnoses on a 7-point Likert scale (1 - Extremely Unlikely, 2, 3, 4 - Neither Likely or Unlikely, 5, 6, 7 - Extremely Likely, 9 - I Don’t Know Enough About This Diagnosis To Answer)? Your answer must be a whole number between 1 and 7. Please include just the diagnosis and your numeric response.

Adjustment Disorder

Generalized Anxiety Disorder

Major Depressive Disorder

Obsessive Compulsive Disorder

Post-Traumatic Stress Disorder

Substance Use Disorder

No Diagnosis

Of the following diagnoses, which do you feel is most likely to be the primary diagnosis that you would want to prioritize as an initial treatment target for Laura? Please select the primary diagnosis and include that below with no other information:

Adjustment Disorder

Generalized Anxiety Disorder

Major Depressive Disorder

Obsessive Compulsive Disorder

Post-Traumatic Stress Disorder

Substance Use Disorder

No Diagnosis

How appropriate do you feel the following treatment models are for addressing Laura’s symptom presentation on a 7-point Likert scale (1 - Extremely Inappropriate, 2, 3, 4 - Somewhat Appropriate, 5, 6, 7 - Extremely Appropriate; 9 - I Don't Know Enough To Answer)? Your answer must be a whole number between 1 and 7. Please include just the treatment and your numeric response.

Cognitive Processing Therapy

Dialectical Behavioral Therapy

Exposure and Response Prevention

Motivational Interviewing

Psychodynamic Psychotherapy

Other Treatment Model (If you selected 'other', please provide more information here on the model you chose in addition to your rating)

Of the following, which treatment model do you feel is the most clinically appropriate to address Laura’s symptom presentation outlined above? Please select the most clinically appropriate treatment model and include that below with no other information:

Cognitive Processing Therapy

Dialectical Behavioral Therapy

Exposure and Response Prevention

Motivational Interviewing

Psychodynamic Psychotherapy

Other Treatment Model (If you selected 'other', please provide more information here)

Please review the following case description:

“David, a 34-year-old white male, has recently begun receiving treatment at an outpatient clinic. He has sought treatment because of self-reported persistent difficulties with substance abuse involving alcohol. This is the first time he has sought treatment. During your risk assessment, he notes that he experiences mostly passive suicidal ideation, but he has no plan to harm himself. During your screen for traumatic exposure, David reports that he was in a serious car accident last year that left him with lingering physical injuries. David has noted that his drinking has led to declining performance at work. He reports consuming between 6-8 12oz. cans of beer each day for the past several years. When he is under the influence of alcohol, he can become irritable and he often initiates arguments with others. After these incidents, he reports feeling remorse and guilt. These events have put a strain on his relationships with his family and friends, and he reports partially seeking treatment to improve his relationships with them. Over the past few years, David has noted that he is often late for work after drinking the previous night. During your evaluation, David reports that he gets very upset easily and subsequently isolates himself.”

Based on the case description above, how likely do you think David is to meet criteria for the following mental health diagnoses on a 7-point Likert scale (1 - Extremely Unlikely, 2, 3, 4 - Neither Likely or Unlikely, 5, 6, 7 - Extremely Likely, 9 - I Don’t Know Enough About This Diagnosis To Answer)? Your answer must be a whole number between 1 and 7. Please include just the diagnosis and your numeric response.

Adjustment Disorder

Generalized Anxiety Disorder

Major Depressive Disorder

Obsessive Compulsive Disorder

Post-Traumatic Stress Disorder

Substance Use Disorder

No Diagnosis

Of the following diagnoses, which do you feel is most likely to be the primary diagnosis that you would want to prioritize as an initial treatment target for David? Please select the primary diagnosis and include that below with no other information:

Adjustment Disorder

Generalized Anxiety Disorder

Major Depressive Disorder

Obsessive Compulsive Disorder

Post-Traumatic Stress Disorder

Substance Use Disorder

No Diagnosis

How appropriate do you feel the following treatment models are for addressing David’s symptom presentation on a 7-point Likert scale (1 - Extremely Inappropriate, 2, 3, 4 - Somewhat Appropriate, 5, 6, 7 - Extremely Appropriate; 9 - I Don't Know Enough To Answer)? Your answer must be a whole number between 1 and 7. Please include just the treatment and your numeric response.

Cognitive Processing Therapy

Dialectical Behavioral Therapy

Exposure and Response Prevention

Motivational Interviewing

Psychodynamic Psychotherapy

Other Treatment Model (If you selected 'other', please provide more information here on the model you chose in addition to your rating)

Of the following, which treatment model do you feel is the most clinically appropriate to address David’s symptom presentation outlined above? Please select the most clinically appropriate treatment model and include that below with no other information:

Cognitive Processing Therapy

Dialectical Behavioral Therapy

Exposure and Response Prevention

Motivational Interviewing

Psychodynamic Psychotherapy

Other Treatment Model (If you selected 'other', please provide more information here)
