# Supplementary material for: Comparing Generative Artificial Intelligence and Mental Health Professionals for Clinical Decision-Making With Trauma-Exposed Populations: Vignette-Based Experimental Study
Source: JMIR Ment Health. 2025 Oct 14;12:e80801. doi: 10.2196/80801 (PMC12527320; doi:10.2196/80801)
Supplement: Multimedia Appendix 2 [file mental-v12-e80801-s002.docx]

**Table S1.** *Alignment of symptoms within vignettes with DSM-5-TR criteria*

| **Disorder** | **DSM-5-TR Diagnostic Criteria** | **Symptoms** |
| --- | --- | --- |
| **Obsessive-Compulsive Disorder (OCD)** | - Recurrent and persistent thoughts, urges or images that are experienced, at some time during the disturbance, as intrusive, unwanted, and that in most individuals cause marked anxiety or distress. | - Excessive fear of accidentally sending inappropriate messages to others  - Excessive fear of someone breaking into home |
|  | - The individual attempts to ignore or suppress such thoughts, urges, or images, or to neutralize them with some thought or action (i.e., by performing a compulsion). | - Compulsive checking of emails and texts prior to sending  - Compulsive checking of locks |
|  | The obsessions or compulsions are time consuming (e.g., take more than 1 hour per day) or cause clinically significant distress or impairment in social, occupational, or other important areas of functioning. | - Engagement in obsessions and compulsions for hours each day |
| **Substance Use Disorder (SUD)** | - Substance is often taken in larger amounts or over a longer period than was intended. | - Consumes 4-6 (female) or 6-8 (male) drinks daily for the past several years |
|  | - Continued substance use despite having persistent or recurrent social or interpersonal problems caused or exacerbated by the effects of substance. | - When under the influence of alcohol, they can become irritable and often initiate arguments with others.  - These events have put a strain on [their] relationships with family and friends. |
|  | - Recurrent substance use resulting in a failure to fulfill rote obligations at work, school, or home. | - Declining work performance and social isolation due to drinking |
