# Supplementary material for: Comparing Generative Artificial Intelligence and Mental Health Professionals for Clinical Decision-Making With Trauma-Exposed Populations: Vignette-Based Experimental Study
Source: JMIR Ment Health. 2025 Oct 14;12:e80801. doi: 10.2196/80801 (PMC12527320; doi:10.2196/80801)
Supplement: Multimedia Appendix 3 [file mental-v12-e80801-s003.docx]

**Table S2.** *Forced-choice selection of diagnosis and treatment options by GAI models.*

| **Outcome** | **No Trauma**  **N (%)** | | | | **Physical Trauma**  **N (%)** | | | | **Sexual Trauma**  **N (%)** | | | |
| --- | --- | --- | --- | --- | --- | --- | --- | --- | --- | --- | --- | --- |
|  | **ChatGPT** | **Claude** | **Gemini** | **Llama** | **ChatGPT** | **Claude** | **Gemini** | **Llama** | **ChatGPT** | **Claude** | **Gemini** | **Llama** |
| **OCD Case** | | | | | | | | | | | | |
| Target Diagnosis | 80  (100%) | 80  (100%) | 80  (100%) | 80  (100%) | 40  (100%) | 40  (100%) | 40  (100%) | 40  (100%) | 40  (100%) | 40  (100%) | 40  (100%) | 25 (62.50%) |
| PTSD Diagnosis | 0  (0%) | 0  (0%) | 0  (0%) | 0  (0%) | 0  (0%) | 0  (0%) | 0  (0%) | 0  (0%) | 0  (0%) | 0  (0%) | 0  (0%) | 15 (37.50%) |
| Target Treatment | 80  (100%) | 80  (100%) | 80  (100%) | 80  (100%) | 40  (100%) | 40  (100%) | 40  (100%) | 40  (100%) | 40  (100%) | 40  (100%) | 40  (100%) | 26 (65.00%) |
| PTSD Treatment | 0  (0%) | 0  (0%) | 0  (0%) | 0  (0%) | 0  (0%) | 0  (0%) | 0  (0%) | 0  (0%) | 0  (0%) | 0  (0%) | 0  (0%) | 14 (35.00%) |
| **SUD Case** | | | | | | | | | | | | |
| Target Diagnosis | 80  (100%) | 80  (100%) | 80  (100%) | 80  (100%) | 40  (100%) | 40  (100%) | 40  (100%) | 40  (100%) | 40  (100%) | 40  (100%) | 40  (100%) | 38 (95.00%) |
| PTSD Diagnosis | 0  (0%) | 0  (0%) | 0  (0%) | 0  (0%) | 0  (0%) | 0  (0%) | 0  (0%) | 0  (0%) | 0  (0%) | 0  (0%) | 0  (0%) | 2  (5.00%) |
| Target Treatment | 80  (100%) | 80  (100%) | 80  (100%) | 80  (100%) | 40  (100%) | 40  (100%) | 40  (100%) | 40  (100%) | 40  (100%) | 40  (100%) | 40  (100%) | 38 (95.00%) |
| PTSD Treatment | 0  (0%) | 0  (0%) | 0  (0%) | 0  (0%) | 0  (0%) | 0  (0%) | 0  (0%) | 0  (0%) | 0  (0%) | 0  (0%) | 0  (0%) | 2  (5.00%) |

*Note*. Forced-choice selection of diagnosis and treatment options in response to vignettes. Target indicates the specific diagnosis or treatment most appropriate for the clinical presentation in the vignette. OCD: Obsessive-Compulsive Disorder; SUD: Substance Use Disorder, PTSD: Post-Traumatic Stress Disorder.
